# Application of infraorbital rim augmentation with pocket fat filling to correct tear trough deformity

**DOI:** 10.3389/fsurg.2023.1112402

**Published:** 2023-05-22

**Authors:** Yukun Liu, Yi Wang, Changqi Cai, Xiuying Wu, Haiping Wang

**Affiliations:** Department of Plastic and Cosmetic Surgery, Tongji Hospital, Tongji Medical College, Huazhong University of Science and Technology, Wuhan, China

**Keywords:** tear trough deformity, fat injection, facial plastic and reconstructive surgery, cosmetic (plastic) surgery, infraorbital rim

## Abstract

**Background:**

Tear trough deformity is one of the most common complaints in clinical settings. The correction of this groove is challenging in facial rejuvenation. The lower eyelid blepharoplasty varies with different conditions. A novel approach of using orbital fat in the lower eyelid to increase the volume of the infraorbital rim with granule fat injection has been applied in our institution for more than 5 years.

**Objectives:**

This article aims to describe the detailed steps of our technique and verify its effectiveness by a cadaveric head dissection after surgical simulation.

**Methods:**

In this study, a total of 172 patients with tear trough deformity underwent lower eyelid orbital rim augmentation with fat filling in the sub-periosteum pocket. According to Barton's grades, 152 patients underwent lower eyelid orbital rim augmentation with orbital fat filling, 12 patients had it combined with autologous granule fat from other body parts, and 8 patients received only transconjunctival fat removal to correct tear trough.

**Results:**

The modified Goldberg score system was used to compare preoperative and postoperative photographs. Patients were satisfied with the cosmetic results. Excessive protruding fat was released, and the tear trough groove was flattened by using autologous orbital fat transplantation. The lower eyelid sulcus deformities were well-corrected. To further illustrate the anatomical structure of the lower eyelid area and injection layers, six cadaveric heads were used for surgical simulation and demonstrated the effectiveness of our technique.

**Conclusions:**

This study indicated that the infraorbital rim could be increased by transplanting orbital fat to the pocket, which was dissected under the periosteum, and the procedure has been verified as reliable and effective.

**Evidence-based medicine (EBM) level:**

Level II.

## Introduction

The term “tear trough deformity” was coined by Flowers in 1993 (1). This deformity is caused by aging or a result of inherited anatomic development (2). The laxation of lacrimal ligament and muscles, dermatolysis, protruding orbital fat, facial volume decrease, and descent of the malar fat pad contributes to tear trough deformity (3). More importantly, the congenital dysplasia of malar complex or bony depression of the orbital rim in the midface due to aging plays an important role in this deformity (4).

A variety of surgical approaches have been proposed to address this deformity with the most popular techniques including hyaluronic acid filling or autologous fat grafting (5). It is not until 1995 that Hamra had come up with the arcus marginalis release and orbital fat preservation, which was regarded as another option for orbital fat removal (6). The utilization of orbital fat to correct tear trough deformity was then modified and improved by other plastic surgeons in recent decades, such as the transconjunctival orbital fat repositioning by Goldberg (7).

However, current techniques have some disadvantages. For instance, orbital fat repositioning could occur with incomplete orbital fat release or unstable fat pedicles fixation. The roof layer was usually regarded as the filling layer for tear trough deformity, but fillers or fat in this layer could move with the orbicularis oculi muscle, thus forming an unattractive appearance. To restore the orbital rim volume is of importance in tear trough correction. Therefore, in this study, we will introduce a new surgical technique which emphasizes the filling in the orbital rim with granule fat in the pocket to correct tear trough deformity.

## Materials and methods

### Patients

From 1 January 2017 to 30 September 2022, a total of 172 patients underwent transconjunctival fat removal followed with or without resected fat grafting at our institution. According to the Barton's grading system, patients were classified based on the severity of their tear trough deformity. The preoperative morphologic characteristics were shown in Table 1. Among these patients, 152 patients underwent lower eyelid orbital rim augmentation with orbital fat filling, 12 patients had it combined with autologous granule fat from other body parts, and 8 patients received only transconjunctival fat removal to correct tear trough. All patients signed the informed consent. Patients were followed up during the first 7 days, then at 3rd month, 6th month, and 1 year postoperatively. Preoperative and postoperative photographs were collected for evaluation.

**Table 1:** Preoperative morphologic characteristics/variations in operative management and satisfaction.

Anatomic analysis	No. of patients	Operation method	No. of patients	Customer satisfaction
Barton's grade 0	8 (4.7%)	Only lower eyelid blepharoplasty	8 (4.7%)	5 (82%)
Barton's grade I	89 (51.7%)	Combined with autologous fat transplantation	152 (88.3%)	147 (96.7%)
Barton's grade II	63 (36.6%)	Combine with autologous fat transplantation		
Barton's grade III	12 (7%)	Combined with extra fat filling	12 (7%)	11 (91.7%)

### Surgical technique

The technique starts with marking the bulging and groove area in the lower eyelid when the patient was sitting and looking straightly ahead. The operation was performed with patients under local anesthesia (1% lidocaine with 1:200,000 epinephrine). Then, a 10-mm incision was made through the tarsus using a no. 11 sharp blade 1.5 mm away from the base of the eyelash that is parallel to the lower eyelid when the patient looked upward in the direction of the head. A blunt dissection was made to expose the orbital septum and remove the excessive orbital fat. We usually excise the central and lateral fat pad, and the medial fat pad is optional, depending on the patient's condition. The resected fat was soaked in 0.9% saline. After the enough amount of fat was harvested, the divided capsulopalpebral fascia and conjunctiva were returned to their normal position and with continuous suture using 7–0 nylon.

**Figure 1:**
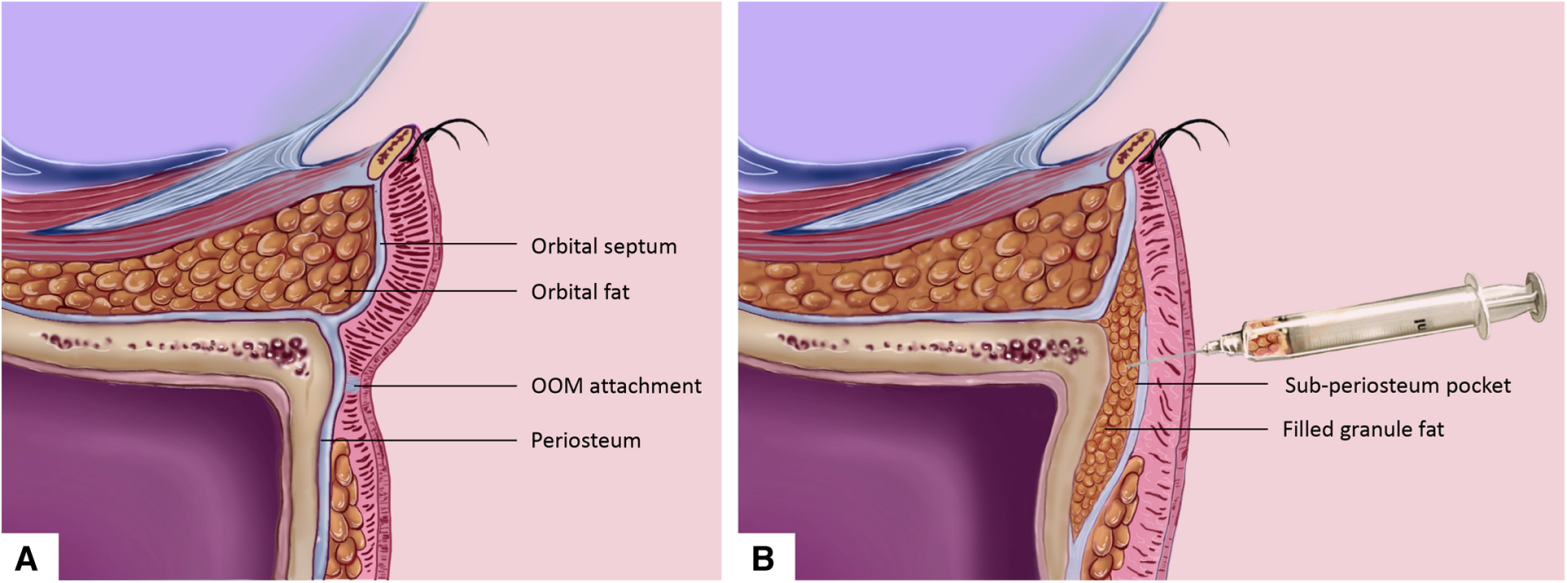
Orbital rim augmentation with fat injection into the sub-periosteum plane for the correction of tear trough deformity. (**A**) Sagittal plane anatomy illustration demonstrates the lower eyelid structure for patient with protruding orbital fat and orbicularis oculi muscle attachment in the tear trough area. (**B**) Excessive orbital fat was taken from the orbital septum by transconjunctival approach. Fat was injected to the sub-periosteum plane by periosteum dissection. OOM, orbicularis oculi muscle.

The resected fat was minced by scissors until it could pass through the injection cannula. Then the fat was transferred into a 1 ml Luer lock syringe for fat injection. The entry point was made in the intersection of the vertical line of the lateral canthus and the horizontal line of the tear trough groove. Local anesthesia was applied in the injection area. We used a 1.2-mm Tulip blunt tip injector to bluntly separate the sub-periosteum pocket in the area around tear trough. Dissection was performed upwardly along the surface of the orbital and maxillary bone under the periosteum, which is about 3 mm superior surpass the infraorbital rim (Figure 1). Our purpose was to release the tear trough ligament thoroughly. Blunt dissection was conducted by an injector needle surpassing the infraorbital rim and closely along the surface of orbital bone, thereby, to create a pocket that is available for fat filling. After the pocket was ready, the target area was compressed for 2 min to prevent hematoma. Then, the prepared fat was injected by an injector in the sub-periosteum layer to create a smoother lid–cheek contour over the groove. If the adipose volume is insufficient to correct the tear trough deformity, fat from other body parts such as the thigh could be used as a donor site. Lower lid skin excision could be used for elderly patients with dermatolysis.

## Results

A total of 172 patients [mean age: 38.73 years; range, 25–59 years; 165 (95.9%) female] underwent operations to correct tear trough deformity. The mean follow-up time was 13.66 months (range, 1–60 months). Of the 172 patients, 152 patients underwent lower eyelid orbital rim augmentation with orbital fat filling, 12 patients had it combined with autologous granule fat from other body parts, and 8 patients received only transconjunctival fat removal to correct tear trough. All procedures were performed by the same surgeon. A total of 163 patients (94.8%) were satisfied with the postoperative outcomes, and most of the patients (*n* = 147, 96.7%) with Barton's grade I and II were satisfied with the outcomes of lower eyelid orbital rim augmentation with orbital fat filling (Table 1).

**Figure 2:**
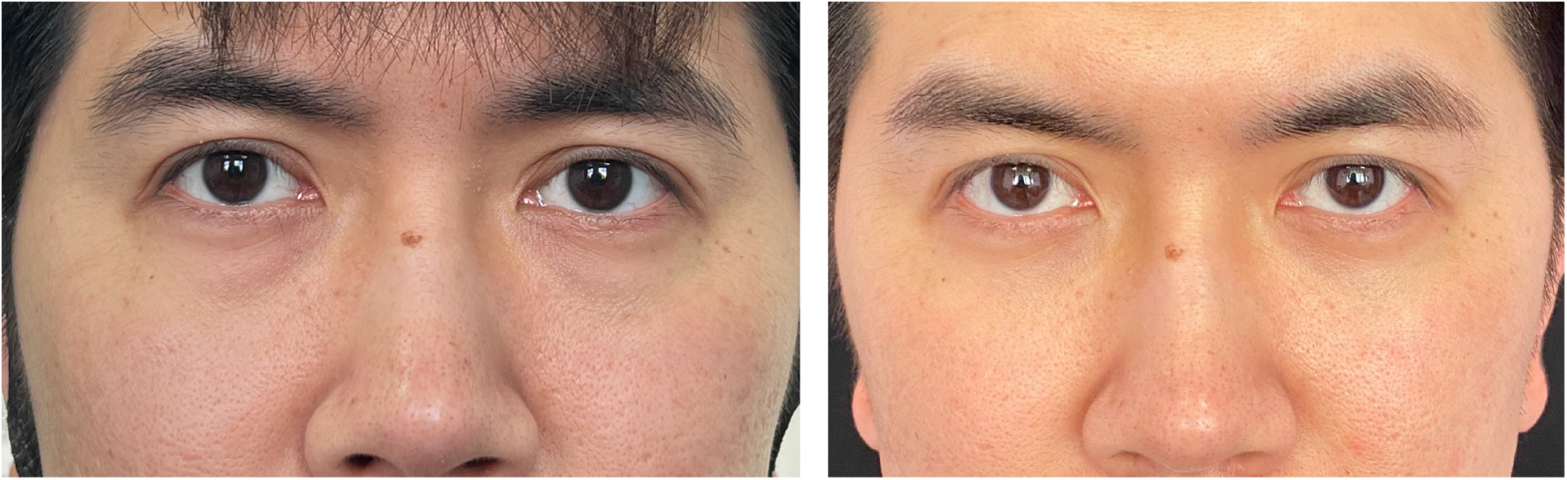
Esthetic result. (**Left**) Preoperative view of a 32-year-old man who underwent infraorbital rim augmentation with pocket fat filling. (**Right**) Six months postoperative view.

**Figure 3:**
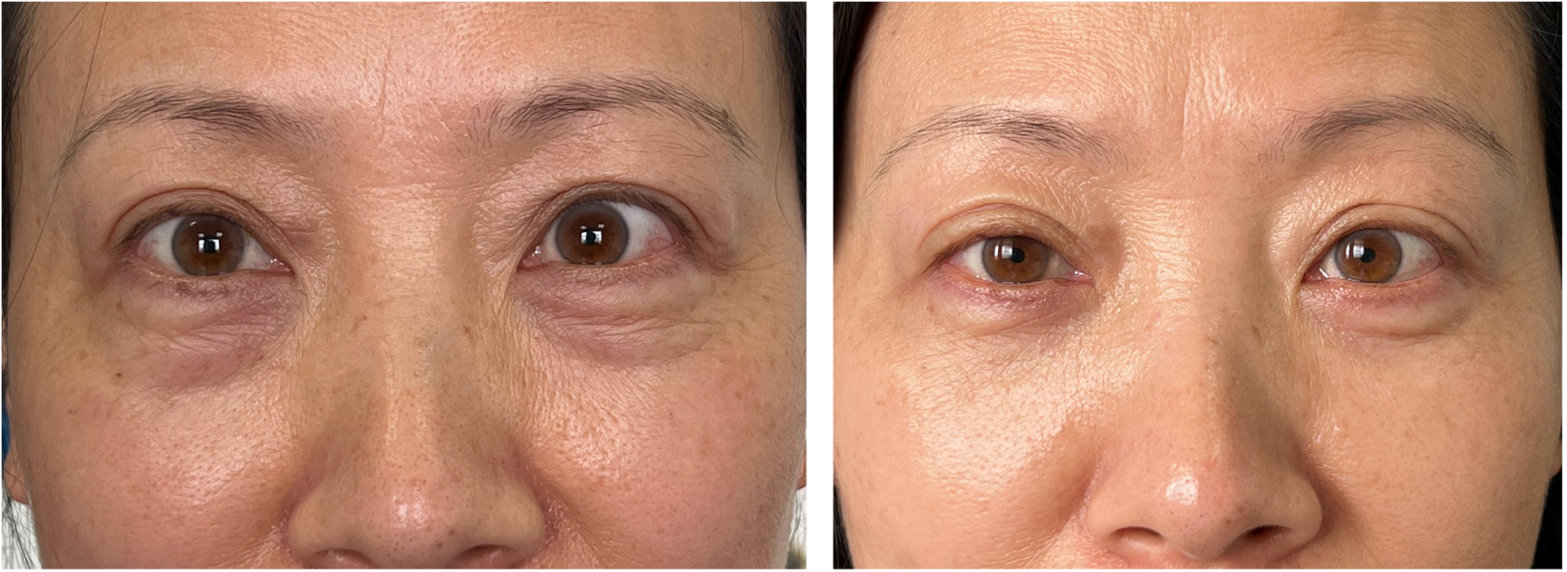
Esthetic result. (**Left**) Preoperative view of a 46-year-old woman who underwent infraorbital rim augmentation with pocket fat filling. (**Right**) One year postoperative view.

**Figure 4:**
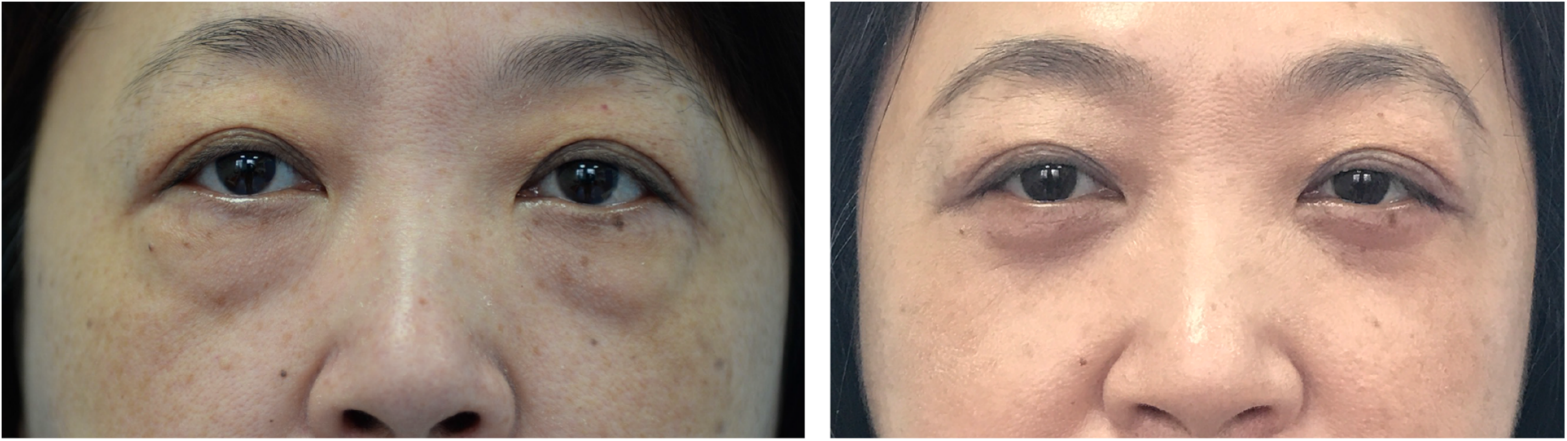
Esthetic result. (**Left**) Preoperative view of a 55-year-old woman who underwent infraorbital rim augmentation with pocket fat filling. (**Right**) Two years postoperative view.

**Figure 5:**
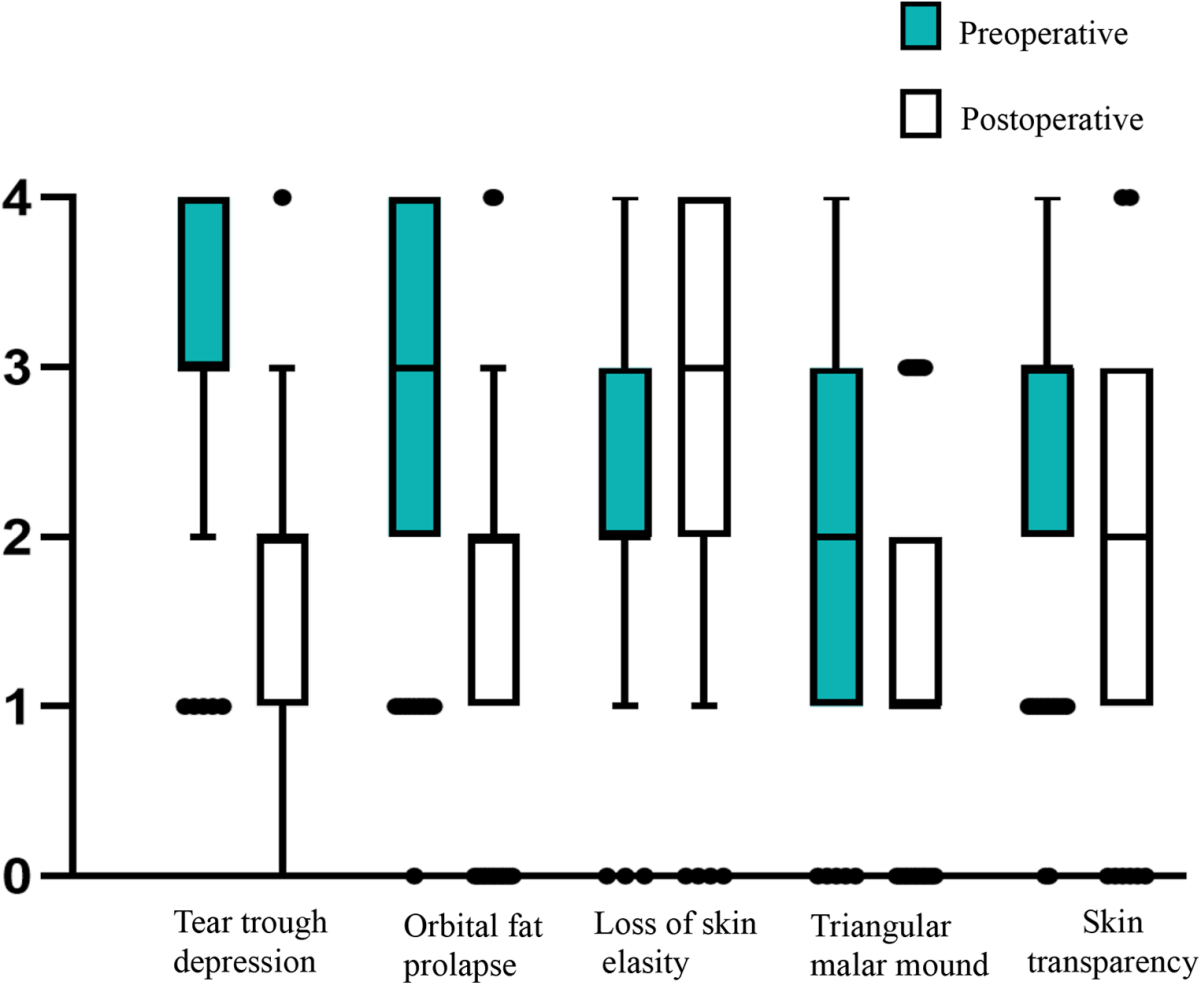
Changes in the mean modified Goldberg scores after transconjunctival fat removal and granule fat injection. Statistical significance was evaluated using a paired, two-tailed *t*-test (*P* < 0.05). Error bars extend to the least and greatest values excluding the outliers.

Preoperative and postoperative photographs of three representative cases are shown in Figures 2–4. Preoperative and postoperative photographs were compared using the modified Goldberg score system. The results indicated improvements in the preoperative and postoperative scores in terms of tear trough depression, orbital fat prolapse, loss of skin elasticity, triangular malar mound, and skin transparency (Figure 5).

Local complications included increased fine wrinkles, transient darkening of the lower eyelids, under-correction of the tear trough, transient infraorbital pain, infraorbital hollowness, infraorbital hardening of subcutaneous nodules, conjunctival granuloma at the incision site, and transient bloodshot eyes (Table 2). The under-correction of tear trough may be due to insufficient pocket fat filling, which can be solved by later filling at least 3 months after. The infraorbital pain and subcutaneous nodules subsided within 1 month. Transient fine wrinkles usually disappear after several months or can be treated by adjuvant laser therapy or mesoderm therapy. The darkening of the lower eyelids may be caused by decreased fat volume or hyperpigmentation after inflammation caused by the operation. However, as we observed, most of the phenomenon fades away in 3 months.

**Table 2:** Complications after transconjunctival fat removal and granule fat injection.

Complications	No. of patients (%) (*n* = 172)
Transient increased fine wrinkles	66 (41.8%)
Transient darkening of the lower eyelids	58 (33.7%)
Under-correction of tear trough	22 (12.8%)
Transient infraorbital pain	14 (11.3%)
Infraorbital hollowness	3 (1.9%)
Infraorbital hardening of subcutaneous nodule	5 (3.1%)
Conjunctival incision site granuloma	2 (1.3%)
Transient bloodshot eye	2 (1.3%)

**Figure 6:**
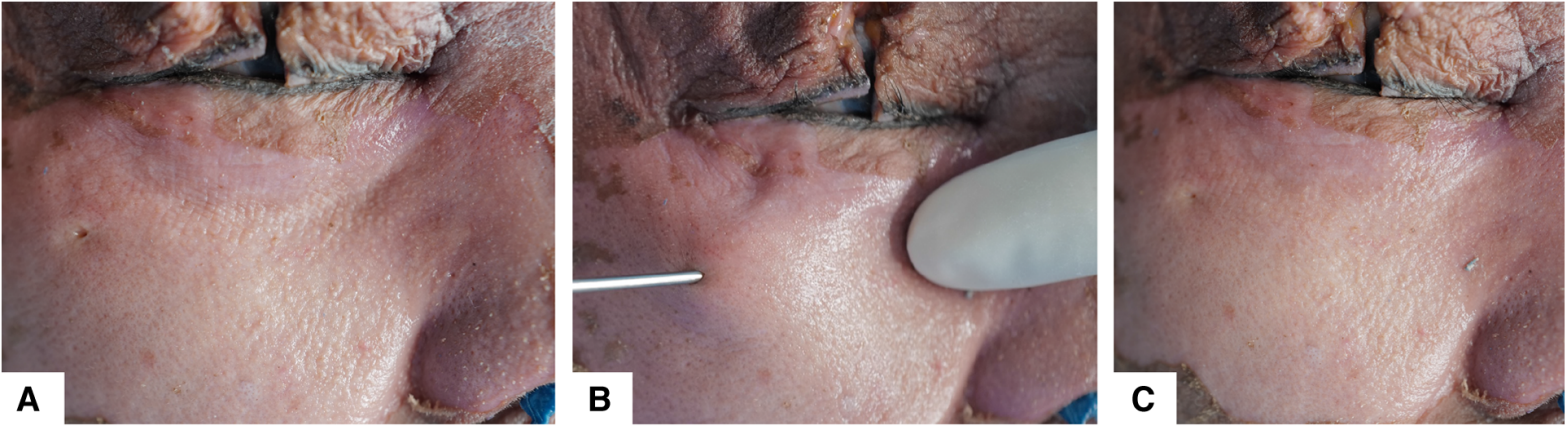
Surgical simulation on cadaveric head. (**A**) Preoperative view with tear trough deformity. (**B**) Intraoperative view, fat filling by a 1.2-mm blunt tip injector into the sub-periosteum plane. (**C**) Postoperative view showed that the lacrimal groove has been corrected.

**Figure 7:**
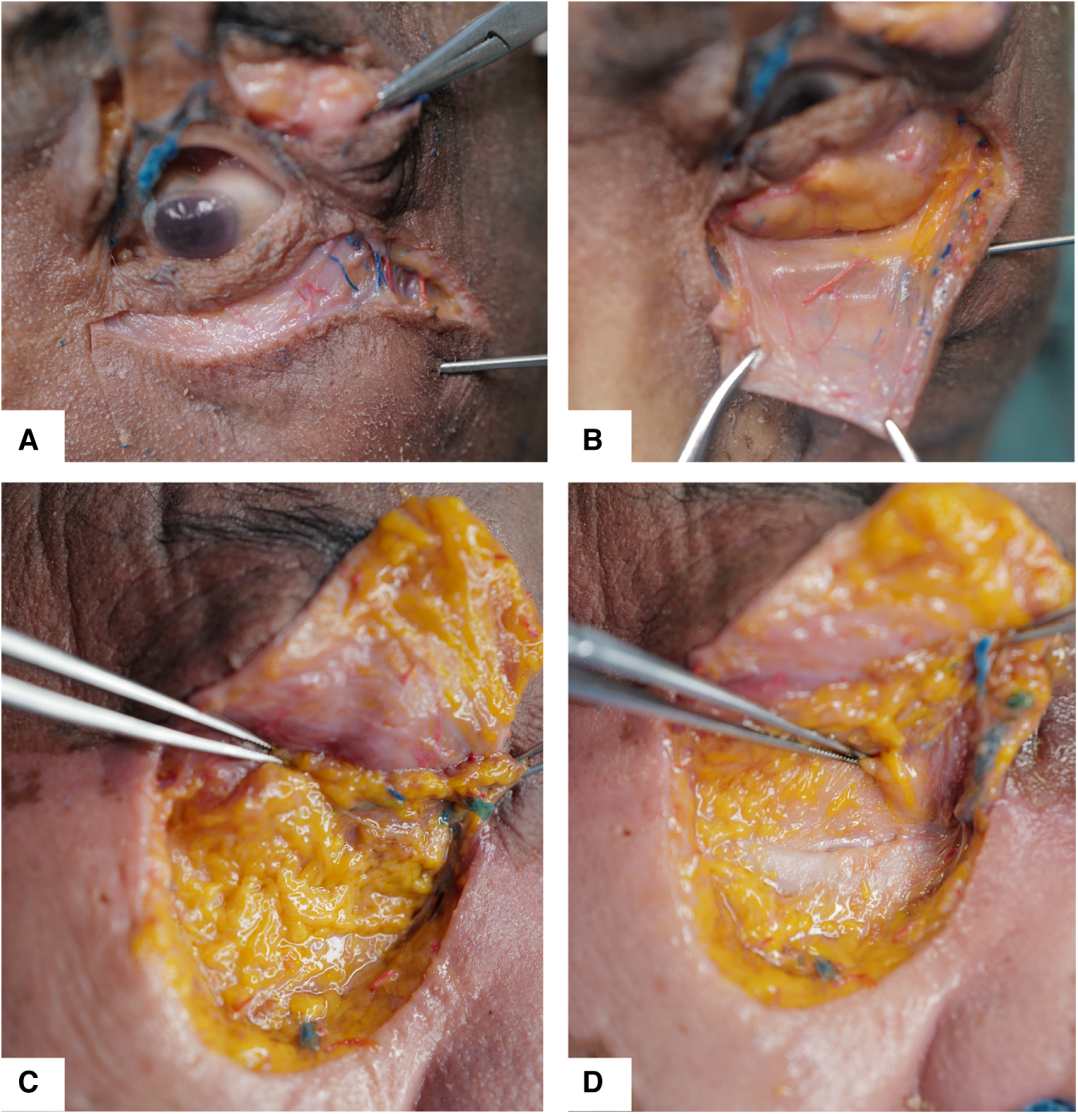
Anatomy layers of lower eyelid was dissected and shown after surgical simulation. (**A**) The incision was made near the lower eyelid margin to elevate the skin muscle flap. (**B**) The orbital fat with septum was exposed, and septum was integrated after the pocket was ready. (**C**) Periosteum was dissected and elevated, and the filled granule fat was seen. (**D**) The tear trough area was covered by filled fat.

To prove the effectiveness of our technique, cadaveric head anatomy and surgical simulation were processed in our institution. Surgical simulation was conducted according to steps introduced in our technique. After operation, incision was made in the midface until the surface of the maxillary and orbital rim. The cadaveric dissection showed a thorough release of orbicularis oculi muscle from orbital bone in the tear trough area. Anatomy verification has demonstrated the filled granular fat in the orbital rim and around the tear trough area (Figures 6, 7). This cadaveric study followed the Declaration of Helsinki.

## Discussion

Tear trough deformity leads to a tired and unattractive appearance. Previous studies have reported many techniques to correct this groove, including skeletal tear implants, infraorbital fat release and repositioning, and fat grafting as well as non-surgical approaches (2). Dermal filling materials such as hyaluronic acid, collagen, poly-L-lactic acid, or calcium hydroxyapatite have been used for tear trough rejuvenation as a non-surgical procedure that is less invasive and with better satisfaction (5, 8). The infraorbital fat was usually repositioned into the supraperiosteal or subperiosteal plane or under the orbicularis oculi muscle for improving the under-eye appearance (9). However, fat release or preservation may be associated with complications such as fat retraction or incorrect evaluation for deficient volume. Moreover, according to our experience, the surgical procedure is complicated and time-consuming, with the risk of an unsmooth curve in the midface and the possibility of a protruding appearance in undesired area postoperatively. Although filling materials spring up currently, with its convenience of achieving immediate effect after injection, they may also have the disadvantages of prolonged edema, Tyndall effect, nodule formation, or the demand of repeat injection (10). Previous literature has demonstrated the effectiveness of autologous fat grafting for tear trough deformity (11). Unlike other filling materials, fat comes from autologous tissue, with the advantages of a less invasive procedure and better tissue compatibility (12). In this technique, autologous fat was harvested from orbital fat, simultaneously addressing the protruding appearance in the lower eyelid region.

In the junction of the orbital region and the midface, tear trough deformity was formed by bony hypoplasia or bony absorption of the orbital rim owning to aging (13). Physiologically, the orbital septum and periosteum are continuous. The orbital fat prolapse and orbital rim hollow are usually combined factors to patients with tear trough deformity (14). People with better orbit rim development have less possibility to show lower eyelid bag. Previous studies have demonstrated an age-related retrusion in the bony orbit (4). The orbital hollow is attributed to volume loss, such as orbital bony absorption in orbicularis oculi muscle attachments, malar fat pad descent, or tissue atrophy (15). The anatomy factor may exist in the ligament structure; however, the existence of tear trough ligament is controversial in previous literatures. We do see the ligament-like fascia; it is regarded as a ligamentous attachment, and most of the attachment is muscle-derived. They usually originated from the orbicularis oculi muscle rather than from the skin. The usually called “lacrimal ligament” could be defined as the sandwiched constructure in between the palpebral and the orbital rim of the orbicularis oculi as combined attachment (3). Lacrimal ligament can be seen as follows: tear trough ligament inferomedially, which continues inferolaterally as the orbicularis retaining ligament (16, 17). Therefore, releasing the attachment but not the ligament in the medial orbital rim is necessary to create an effective space for fat filling. The surgical release of the attachment in the tear trough area and graft with orbital fat was effective to solve the groove. Moreover, the release of tear trough attachment has been demonstrated to not weaken the tarsoligamentous supporting system of the lower eyelid (18).

To achieve the augmentation effect of the orbital rim, our technique emphasized the dissection to form a pocket under the periosteal plane. The dissection was processed between the orbital rim and periosteal plane, surpass the orbital rim upwardly until the superficial surface of the orbital septum and the pocket should be enough for fat grafting to make a more natural and smooth transition. The classical procedure from Hamra, which resected and released the arcus marginalis, preserved fat to address the decrease of volume in the lower eyelid (6). Unlike this procedure, our technique emphasizes that the granular fat filling layer is relatively profound, which is under the periosteum and upwardly close to the surface of the orbital rim. Our technique avoided forming a nodular appearance, which may occur in fat filling under the muscles. The fat that filled in the orbital rim is the supplement of volume decrease, blending the eyelid–cheek junction and supporting the strength of tissue in the midface. Previous studies have reported preferable outcomes in plenty of cases with granule fat filling (19). The abundant blood supply in the midface guarantees the survival rate of the granular fat in this area (20). In order to avoid uneven filling, Su et al. use the two-dimensional fat injection approach, based on previous publication (8). However, our technique just needs single tunnel injection with one entry point on the area under the lateral canthus. The injection should break through the periosteum, and the fat grafting has the advantage of strengthening the front wall of the lower eyelid. The filling should place granule fat in the sub-periosteum evenly around the tear trough groove.

In some patients, the volume of infraorbital fat may not be enough for correcting tear trough. Hence, to achieve better effect, fat from other body parts such as the thigh can be used for supplementary filling. Elderly patients usually need more fat because of more bony absorption and more area in demand of fat injection. In addition, fat filling for lower eyelid is a part of facial fat filling such as malar cheek junction (21). Insufficient correction of tear trough can be resolved by repeated fat filling after 6 months. This technique is also suitable for elderly patients with dermatolysis by removing excessive loose skin after completing all the steps above. When resecting redundant skin, incision was made closely to lower eyelid rim, and the orbicularis oculi muscle should be preserved to suspend on the periosteum in the lateral orbit (22). The individual components of facial fat compartment, orbicularis oculi muscle, ligament system, and lower lid skin are important to achieve successful lower lid blepharoplasty (23). Comparing with the orbital septum release and fat repositioning, our technique kept the septum integrated. We believed that there is more tendency of fat filling and less septum reset in blepharoplasty. Therefore, our technique has the advantages of more physiological, quick recovery and less injury with fewer complications. Less complications may attribute to no artery bleeding found in the area. Compression is effective if the branch of angular vein was injured and bleeding, but no hematoma was observed in our patients. High satisfaction may own to the safe and effective technique resolving the protruding appearance. Study limitations must be addressed. The follow-up time is relatively short for most of our patients, and long-term follow-up longer than 24 months is no more than 20%. Therefore, we were unable to analyze and identify the long-term postoperative outcomes and maintenance duration. In addition, we did not compare the outcomes and effectiveness of this technique with other techniques such as arcus marginalis release and orbital fat preservation (6). Future studies should be conducted with more comparisons with other lower eyelid procedures and longer follow-up time.

## Conclusion

Pocket fat filling were applied to increase the volume of the infraorbital rim to correct tear troughs and palpebromalar grooves, and for some patients, addressing lower eyelid bags. This reliable technique achieves excellent cosmetic outcomes and is associated with high patient satisfaction. We believe that this procedure will continue to be used by surgeons to improve lower eyelid rejuvenation.

## Data Availability

The original contributions presented in the study are included in the article, further inquiries can be directed to the corresponding author.
